# A Novel Antidiabetic Monomers Combination Alleviates Insulin Resistance Through Bacteria-Cometabolism-Inflammation Responses

**DOI:** 10.3389/fmicb.2020.00173

**Published:** 2020-02-18

**Authors:** Lin Han, Lin-Hua Zhao, Ming-Liang Zhang, Hua-Ting Li, Ze-Zheng Gao, Xiao-Jiao Zheng, Xin-Miao Wang, Hao-Ran Wu, Yu-Jiao Zheng, Xiao-Tian Jiang, Qi-You Ding, Hao-Yu Yang, Wei-Ping Jia, Xiao-Lin Tong

**Affiliations:** ^1^Department of Endocrinology, Guang’anmen Hospital, China Academy of Chinese Medical Sciences, Beijing, China; ^2^Laboratory of Molecular and Biology, Guang’anmen Hospital, China Academy of Chinese Medical Sciences, Beijing, China; ^3^Department of Endocrinology and Metabolism, Shanghai Jiao Tong University Affiliated Sixth People’s Hospital, Shanghai, China; ^4^Beijing University of Chinese Medicine, Beijing, China; ^5^Center for Translational Medicine, and Shanghai Key Laboratory of Diabetes Mellitus, Shanghai Jiao Tong University Affiliated Sixth People’s Hospital, Shanghai, China; ^6^Department of Endocrinology, Affiliated Hospital of Changchun University of Chinese Medicine, Changchun, China

**Keywords:** antidiabetic monomer combination, insulin resistance, bacteria, cometabolism, inflammation

## Abstract

The present study sought to examine the therapeutic effect of a novel antidiabetic monomer combination (AMC) in treating type 2 diabetes mellitus (T2DM); while also elucidating the potential functional mechanism. Male C57BL/6J mice were fed a high-fat diet (HFD) for 12 weeks to establish T2DM. The AMC group showed significant reduction in weight, fasting blood glucose (FBG), serum total cholesterol (TC) and low density lipoprotein cholesterol (LDL-C), and experienced reduced insulin resistance based on oral glucose tolerance testing (OGTT) and hyperinsulinemic-euglycemic clamp testing (“gold standard” for determining *in vivo* insulin sensitivity). Further, AMC restored the altered intestinal flora by increasing the abundance of the beneficial bacteria *Akkermansia*, and decreasing the number of harmful bacteria, including *Bacteroides*, *Odoribacter*, *Prevotella 9*, *Alistipes*, and *Parabacteroides*. Components of the host-microbial metabolome were also significantly changed in the AMC group compared to the HFD group, including hydroxyphenyllactic acid, palmitoleic acid, dodecanoic acid, linoleic acid, and erucic acid. Furthermore, AMC was found to inhibit inflammation and suppress signaling pathways related to insulin resistance. Lastly, spearman correlation analysis revealed relationships between altered microbial community and co-metabolite levels, co-metabolites and inflammatory cytokines. Hence, the potential mechanism responsible for AMC-mediated alleviation of insulin resistance was suggested to be involved in modulation of bacteria-cometabolism-inflammation responses.

## Introduction

Insulin resistance plays a critical role in the development of type 2 diabetes mellitus (T2DM) (Liu B. et al., 2017) and is a characteristic feature of the associated clinical metabolic disorders that include obesity, metabolic syndrome, and cardiovascular disease (Barchetta et al., 2019; Friesen and Cowan, 2019). Many studies have also confirmed an association between insulin resistance and chronic inflammation (Chan et al., 2017; Zhang et al., 2017; Bao et al., 2018).

Gut microbiota is an integral component of the human body, allowing for the exchange of substances and information between microbes and the host, and contributing to host nutrition, metabolism, and immune regulation (Mukherjee et al., 2018; Yang et al., 2018). Hence, in healthy organisms, consumption of high-fat diets (HFD) can significantly alter the balance and structure of intestinal microflora and cause chronic inflammation leading to the development of T2DM (Kahn et al., 2006; Saad et al., 2016).

Traditional Chinese medicine (TCM) has a long history of use for the prevention and treatment of diabetes. Recently, much evidence-based medical data has confirmed the curative effect of TCM on diabetes and associated complications (Liu Q. et al., 2017; Gao et al., 2018; Nie et al., 2019; Xiang et al., 2019). TCM Herbal formulations are guided by the principles of compatibility and of “monarch, minister, assistant, and guide,” which are applied when deciding which combination of herbs will achieve the desired effect in treating the disease (Ma et al., 2019). Gegen Qinlian decoction, a representative TCM prescription from the traditional medical book “Treatise on Febrile Diseases” functions to remove internal heat and relieve exterior syndromes. Thus, it is used to treat fever and diarrhea caused by inward invasion of the greater yang exterior pathogen (Wang et al., 2015; Wu et al., 2019). Professor Xiao-Lin Tong from Guang’anmen Hospital, China Academy of Chinese Medical Sciences reported the beneficial effects of Gegen Qinlian decoction in the treatment of T2DM (Xu J. et al., 2015). Moreover, a modified form of this treatment was developed based on the TCM theory, and exhibited significant clinical hypoglycemic effects. In order to improve quality control and standardization of this prescription, an innovative research approach was designed to allow for the application of TCM in treating disease by combining the primary monomer compounds in a single formula. The present study sought to investigate the effects of combining major monomers in the modified Gegen Qinlian decoction formula on insulin resistance, and subsequently elucidating the mechanism of action in T2DM treatment. The antidiabetic monomer combination (AMC) in the modified Gegen Qinlian decoction was composed of puerarin, baicalin, berberine, mangiferin, paeoniflorin, and ginsenoside-Rb1, which are the active compounds in Radix puerariae (Ge-Gen), Radix scutellariae (Huang-Qin), Rhizoma coptidis (Huang-Lian), Anemarrhenae rhizoma (Zhi-Mu), Radix paeoniae rubra (Chi-Shao), and Panax quinquefolium (Xi-Yang-Shen), respectively.

The efficacy and potential mechanism of action employed by AMC was explored in HFD-induced T2DM mice.

## Materials and Methods

### Experimental Animals and Procedure

Thirty-six male C57BL/6J mice (18–22 g, 7 weeks old) were purchased from Nanjing Biomedical Research Institute of Nanjing University, Nanjing, Jiangsu, China. The care and use of animals were in accordance with the Provisions and General Recommendation of the Chinese Experimental Animals Administration Legislation. All efforts were made to minimize animal suffering. Mice were housed in a specific-pathogen-free (SPF) facility, with climate-controlled rooms maintained at 24 ± 2°C, 60–70% relative humidity, and controlled 12 h light/dark cycles. Nine mice were housed per cage, and all mice had free access to autoclaved food and water. The bedding was autoclaved and changed twice per week. Mice were acclimatized for 1 week before beginning the study. All animal experimentation procedures were performed according to the Chinese Guidelines for Animal Care, which conform with the internationally accepted uses of experimental animals, and the protocols were reviewed and approved by the Animal Ethics Committee of Nanjing Biomedical Research Institute of Nanjing University (the approval number: NRCMM009).

Mice were randomly divided into four groups (*n* = 9) as follows: a control group fed a normal diet (10 kcal% fat) (ND group); a control group fed an HFD (60 kcal% fat) (HFD; D12492, Research Diets, New Brunswick, NJ, United States) (HFD group); metformin (MET) group, mice fed an HFD supplemented with MET [250 mg/kg body weight (BW)/day]; and AMC group, mice fed an HFD supplemented with AMC (composed of 3.72 mg/kg.BW/day puerarin, 60.36 mg/kg.BW/day baicalin, 216.00 mg/kg.BW/day berberine, 8.55 mg/kg.BW/day mangiferin, 35.73 mg/kg.BW/day paeoniflorin, and 6.19 mg/kg.BW/day ginsenoside-Rb1). All drugs were administered to mice by gavage in distilled water, mice were given oral administration in a volume of 0.1 mL/10 g.BW, and treatment frequency is once a day for 12 weeks. ND and HFD groups were administered equal volumes of distilled water to minimize the effect of the gavage procedure.

During the course of the 12-week treatment, body weight was measured once per week, fasting blood glucose (FBG) was monitored every 2 weeks, and fecal samples were collected at week 0, 4, 8, and 12, and stored at -80°C until further analysis. After 12 weeks of treatment, three mice per group were subjected to glucose clamp testing. Subsequently, percent body fat of the remaining mice (*n* = 6 per group) was determined with the PIXImus2 imager (General Electric Lunar, Madison, WI, United States) as per the manufacturer’s instructions, and oral glucose tolerance tests (OGTT) were performed. At the end of the study, after a 12-h fasting period, the remaining mice were euthanized using CO_2_ and the following specimens were harvested for further analyses: blood was collected by cardiac puncture, centrifuged at 1,000 × *g* for 10 min at 25°C, and the serum was collected into fresh tubes; liver tissues were harvested and stored at -80°C for western blot analyses; tissue samples of the small intestine were harvested and fixed in 10% formaldehyde solution for immunohistochemical (IHC) analyses.

### Analyses of Serum Biochemical Indicators

Total cholesterol (TC) and low density lipoprotein cholesterol (LDL-C) content in serum were analyzed using a Hitachi 7020 automatic biochemical analyzer (Hitachi, Tokyo, Japan) as per the manufacturer’s protocol.

### Hyperinsulinemic-Euglycemic Clamp Test

After the 12-week treatment period, three mice per group were subjected to glucose clamp testing. Mice were catheterized 4 days prior to testing as described previously (Hui et al., 2017). At the end of the experiment, mice were sacrificed and the soleus, gastrocnemius, superficial vastus lateralis, epididymal fat, and liver were isolated. The corresponding radioactivity was measured by scintillation.

### Western Blot Analyses of Mice Liver Tissues

Liver tissue samples were lysed in radio-immunoprecipitation assay (RIPA) buffer on ice for 20 min and the lysates were centrifuged at 13,000 × *g* for 10 min at 4°C. Supernatants were collected and the protein concentration was measured using the bicinchoninic acid (BCA) protein assay kit (Cwbiotech, Beijing, China) according to the manufacturer’s instructions. Proteins were separated on 8% sodium dodecyl sulfate polyacrylamide gel electrophoresis (SDS-PAGE), and then transferred onto 0.45 μm polyvinylidene difluoride (PVDF) membranes (Millipore, Billerica, MA, United States). Membranes were blocked with 5% bovine serum albumin-tris-buffered saline with Tween 20 (BSA-TBST) for 60 min at 25°C, washed with TBST, and incubated with the following monoclonal primary antibodies diluted 1:1,000 in 5% BSA-TBST at 4°C overnight: rabbit anti-InsR (3025, CST, Boston, MA, United States), rabbit anti-INRS-2 (ab134101, Abcam, Cambridge, United Kingdom), rabbit anti-p-Akt (4060, CST), rabbit anti-Akt (4691, CST), and rabbit anti-β-actin (TA-09, Zhongshan Jinqiao, Beijing, China). After incubation, membranes were washed and incubated with horseradish peroxidase (HRP)-conjugated goat anti-rabbit IgG secondary antibody diluted 1:1,0000 in 5% BSA-TBST for 40 min at 25°C, washed with TBST, and the protein-antibody complexes were visualized using the enhanced chemiluminescent (ECL) detection kit (Millipore, Billerica, MA, United States) according to the manufacturer’s instructions. Band intensities were quantified using a Gel Image System (ver. 4.00, Tanon, Shanghai, China). Relative protein expression levels of InsR, IRS-2, *p*-Akt, and Akt were normalized to that of β-actin.

### IHC Analyses of Mice Small Intestinal Tissue

The expression level of specific inflammatory cytokines, including tumor necrosis factor-alpha (TNF-α), interleukin- (IL) 1β, and IL-6 in the small intestine tissue samples were detected using IHC. Tissue blocks were embedded in paraffin, sectioned, mounted on slides, dewaxed with xylene, and hydrated using alcohol gradient. Endogenous peroxidase activity was blocked with 3% hydrogen peroxide (H_2_O_2_) in methanol and the tissue sections were incubated with goat serum, and subsequently with the following primary antibodies: mouse-anti-TNF-α (1:50, sc-52746, Santa Cruz Biotechnology, CA, United States), rabbit-anti-IL-1β (1:100, AF5103, Affinity, Cincinnati, OH, United States), and rabbit-anti-IL-6 (1:200, 21865-1-AP, Sanying, Wuhan, China), for 2 h at 37°C. Tissue sections were incubated with HRP-labeled goat anti-rabbit or anti-mouse secondary antibody using EliVision plus IHC kit (Kit-9902, Maixin, Fuzhou, China) for 30 min at 37°C as per the manufacturer’s instructions. Tissue sections were then stained with 3,3′-Diaminobenzidine (DAB), counterstained with hematoxylin, sealed with neutral gum, and dehydrated sequentially using an alcohol gradient. Five high-powered lens fields (400 × magnification) were randomly chosen from each section under a light microscope (Olympus, BX43, Tokyo, Japan) to assess the integrated optimal density (IOD) of TNF-α, IL-1β, and IL-6.

### 16S rRNA Gene Analyses

The 16S sequencing procedure was performed at the Institute of Microbiology, Chinese Academy of Sciences (Beijing, China). DNA was extracted from 0.5 g of mice fecal samples using QIAamp PowerFecal DNA kit (QIAGEN, Germany) according to the manufacturer’s protocol. The V3 and V4 region of the bacterial 16S rDNA gene was amplified by polymerase chain reaction (PCR) using the primers: 341F (5′-CCTACGGGNBGCASCAG-3′) and 805R (5′-GACTACNVGGGTATCTAATCC-3′). All PCR reactions were performed in a total reaction volume of 25 μL containing 5 μL of 5 × GC buffer, 0.5 μL of KAPA dNTP mix, 0.5 μL of KAPA HiFi HotStart DNA polymerase, 0.5 μL of each primer (10 pM), and 50–100 ng of template DNA. The PCR amplification protocol used was as follows: initial denaturation at 95°C for 3 min, followed by 25 cycles at 95°C for 30 s, 55°C for 30 s, and 72°C for 30 s, and final extension at 72°C for 5 min. The 16S V3 and V4 amplicons were purified from free primers and primer dimer species by PCR clean up using AMPure XP beads. The purified product was amplified by PCR using primers, wherein the barcode specifies an eight-base sequence unique to each sample. All PCR reactions were performed in a total volume of 25 μL containing 5 μL of 5 × GC buffer, 0.75 μL of KAPA dNTP Mix, 0.5 μL of KAPA HiFi HotStart DNA polymerase, 1.5 μL of each primer (10 pM), and 5 μL of the purified product. PCR amplification reactions included initial denaturation at 95°C for 3 min, followed by eight cycles at 95°C for 30 s, 55°C for 30 s, and 72°C for 30 s, and final extension at 72°C for 5 min. The amplicons were subsequently purified using AMPure XP beads to clean up the final library before quantification. Purified amplicons were pooled in equimolar and were paired-end sequenced (2 × 250) on an Illumina Hiseq2500 platform according to the standard protocol.

GenBank accession number: The 16S rRNA gene data generated in this study were submitted to NCBI GenBank database under the accession number PRJNA593365.

### Metabolite Measurement

Targeted quantitative metabolomics profiling of fecal samples of both human and mice was performed by Metabo-Profile Biotechonology Co., Ltd., (Shanghai, China). The fecal samples used for analyses were the same as those used for 16S sequencing. Targeted identification and measurement of fecal metabolomics were performed based on a validated method as described previously (Zhao et al., 2017). Briefly, approximately 50 mg of fecal samples was homogenized with 300 μL of NaOH (1M) solution, the residue after centrifuging was further exacted with 200 μL of cold methanol, and the resulting supernatant were combined together. The supernatant samples were then capped and submitted for automated sample derivatization using Methyl chloroformate (MCF) with a robotic multipurpose sampler (MPS) 2 with dual heads (Gerstel, Muehlheim, Germany). Subsequently, the derivatized samples were analyzed and quantitated with a gas chromatography coupled to time-of-flight mass spectrometry (GC-TOFMS) system (Pegasus HT, Leco Corp., St. Joseph, MO, United States), separated on a 5% diphenyl/95% dimethyl polysiloxane column (Rxi-5MS, 30 m length × 250 μm I.D. × 0.25 μm film thickness) and operated in electron ionization (EI) mode. The quality control samples were prepared by the same procedures as the test samples. The raw data generated by GC-TOFMS were processed and analyzed using proprietary software XploreMET (v2.0, Metabo-Profile, Shanghai, China).

### Statistical Analyses

Data are expressed as the mean ± standard error of mean (SEM) for a minimum of three independent experiments. Statistical analyses were performed using the SPSS software (SPSS 20.0, SPSS Inc., Chicago, IL, United States). Two-tailed Student’s *t*-tests, one-way analysis of variance (ANOVA) with Dunnett’s *post hoc* tests, and Games–Howell tests were used to compare the groups. *p* < 0.05 was considered statistically significant. Correlations between metabolite and bacterial compositions, metabolites and inflammatory cytokines were assessed by spearman correlation testing.

## Results

### AMC Treatment Alleviated General Indicators of HFD-Induced Diabetes

**FIGURE 1:**
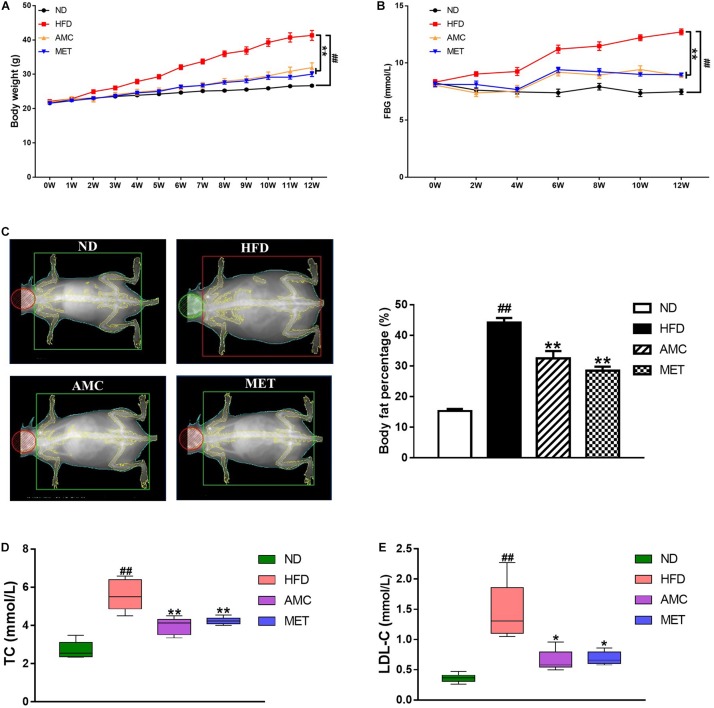
Effects of AMC on general indicators of diabetes induced by 12-week high-fat diet. Body weight **(A)** was monitored once a week, and fasting blood glucose (FBG) **(B)** every 2 weeks during 12-week trial. **(C)** Body fat percentage was measured using bone densitometer at the end of the last week of trial. Total cholesterol (TC) **(D)** and low density lipoprotein cholesterol (LDL-C) **(E)** levels in mice serum were analyzed using an automatic biochemical analyzer. Data are shown as means ± standard error of mean (SEM) of nine **(A,B)** or six **(C–E)** independent experiments and were evaluated by ANOVA with Dunnett’s *post hoc* tests. ^#^*p* < 0.05, ^##^*p* < 0.01, compared with the ND group; *p < 0.05, **p < 0.01, compared with HFD group.

During the 12-week treatment period, the weight of mice in the HFD group continued to increase and was significantly higher than that of the ND group at each time point. However, the weight of mice significantly decreased in the drug-intervention groups. The extent of weight reduction was similar between the AMC and MET groups (Figure 1A).

During the course of the experiment, FBG was found to be significantly higher in the HFD group compared to the ND group. After the 12-week treatment period, AMC and MET intervention achieved significant glucose-reducing effects (Figure 1B).

The body fat percentage was significantly higher in the HFD group compared to that of the ND group. During the course of the 12-week treatment period, intervention with AMC and MET slowed down the HFD-induced increase in body fat content (Figure 1C).

Furthermore, AMC supplement significantly inhibited the increase in serum TC and LDL-C levels in the AMC group compared to that of the HFD group, indicating the protective and preventive effect of AMC in HFD-induced complications (Figures 1D,E).

### AMC Treatment Improved HFD-Induced Insulin Resistance

**FIGURE 2:**
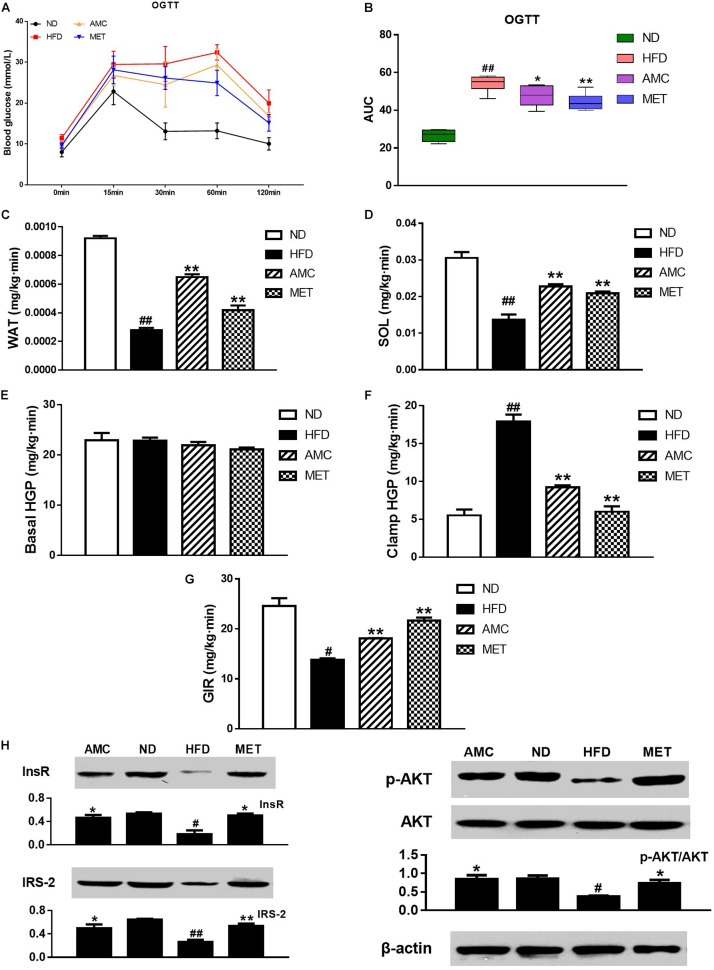
Effects of AMC administration on diet-induced insulin resistance. **(A)** Blood glucose levels in OGTT, and AUC **(B)** is area under the blood glucose-time curves of OGTT. In hyperinsulinemic-euglycemic clamp test, the ability of white adipose tissue (WAT) **(C)** and soleus (SOL) **(D)** in glucose uptake, hepatic glucose output (HGP) in normal **(E)** or high **(F)** insulin state, and glucose infusion rate (GIR) **(G)** were measured, respectively. The protein expressions of InsR, IRS2, p-Akt and Akt **(H)** in liver tissues detected via western blot assay. Data are shown as means ± SEM of six **(A,B)** or three **(C–H)** independent experiments and were evaluated by ANOVA with Dunnett’s *post hoc* tests **(A–F,H)** and Games–Howell tests **(G)**. ^#^*p* < 0.05, ^##^*p* < 0.01, compared with the ND group; *p < 0.05, **p < 0.01, compared with HFD group.

OGTT was used to determine the effect of AMC and MET treatment regimens on glucose metabolism in mice. The results showed significant improvement in glucose clearance by AMC and MET drug-intervention as reflected by the lower glucose peaks. Glucose levels detected by OGTT were similar in both AMC and MET groups (Figure 2A). In OGTT, the glucose area under the curve (AUC) was significantly smaller in the AMC and MET groups compared to the HFD group (Figure 2B), indicating improved glucose tolerance in HFD-induced diabetic mice by AMC and MET treatment.

Hyperinsulinemic-euglycemic clamp is the gold standard for investigating and quantifying insulin resistance. Insulin resistance is a barrier to fat and muscle glucose uptake. Results from hyperinsulinemic-euglycemic clamp testing indicated that AMC-induced enhanced glucose uptake by fat (Figure 2C) and muscles (Figure 2D) in HFD-induced diabetic mice by inhibiting HFD-induced insulin resistance of fat and muscle tissues. Hepatic insulin resistance refers to the decreased ability of insulin to inhibit hepatic glucose production (HGP). These results suggest the absence of significant change in the HGP rate following drug administration in the absence of high levels of insulin (Figure 2E). However, in a hyperinsulinemic condition, AMC significantly inhibited hepatic insulin resistance in HFD-induced diabetic mice (Figure 2F). Further, glucose infusion rate (GIR) is a quantitative indicator of insulin resistance. When hyperinsulinemic-euglycemic clamp testing reached homeostasis, AMC treatment enhanced the glucose infusion rate by raising the overall insulin sensitivity in HFD-induced diabetic mice leading to improved insulin resistance (Figure 2G).

Since the liver is one of the primary target organs of insulin action, levels of key proteins involved in insulin signaling pathway including InsR, IRS2, and phospho-Akt were detected to investigate insulin resistance in all experimental groups. Figure 2H represents the results of western blot analyses for InsR, IRS-2, p-Akt, and Akt expression in the liver tissue following different treatment protocols. The relative expression of InsR and IRS-2 was significantly increased in the drug-intervention (AMC and MET) groups compared to that in the HFD group. In addition, supplementation with AMC and MET enhanced Akt (Ser473) phosphorylation in the liver tissue of HFD-induced diabetic mice.

### AMC Treatment Relieved HFD-Induced Intestinal Inflammation

**FIGURE 3:**
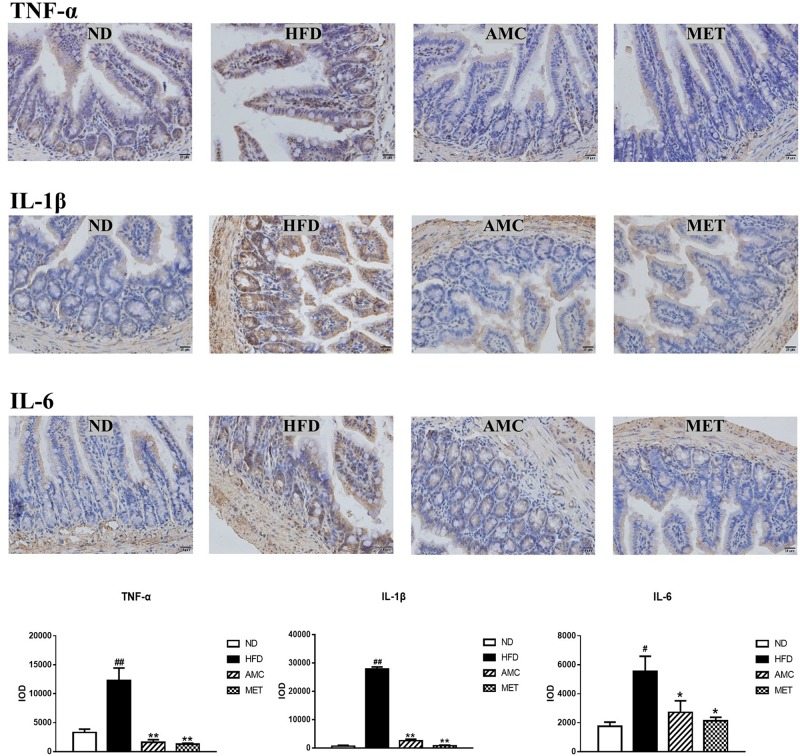
Influence of AMC administration on diet-induced intestinal inflammation. Expression levels of inflammatory cytokines (TNF-α, IL-1β, IL-6) in the small intestinal tissues were determined by IHC. The average optical density of the results was analyzed using image analysis system. Data are shown as means ± SEM of three independent experiments and were evaluated by ANOVA with Dunnett’s *post hoc* tests. ^#^*p* < 0.05, ^##^*p* < 0.01, compared with the ND group; *p < 0.05, **p < 0.01, compared with HFD group.

Differential expression of inflammatory cytokines (TNF-α, IL-1β, and IL-6) in the small intestinal tissue of each experimental group was analyzed by IHC staining. The inflammatory cytokines showed strong cytoplasmic expression in epithelial cells (clear brown color) of the HFD group compared to that of the ND group. The cytoplasmic expression of inflammatory cytokines in the small intestinal epithelial cells was visualized as brown-yellow stained regions in the drug-intervention (AMC and MET) groups compared to the weaker expression seen in the HFD group. The average optical density of IHC results analyzed by the image analyses system indicated significantly higher expression of TNF-α, IL-1β, and IL-6 in the small intestinal tissue of the HFD group compared to the other groups (*p* < 0.01). However, both AMC and MET treatment reduced the expression of inflammatory factors, particularly TNF-α, to levels lower than that observed in the ND group (Figure 3).

### AMC Treatment Induced Dynamic Changes in the Community Structure of Gut Microbiota

**FIGURE 4:**
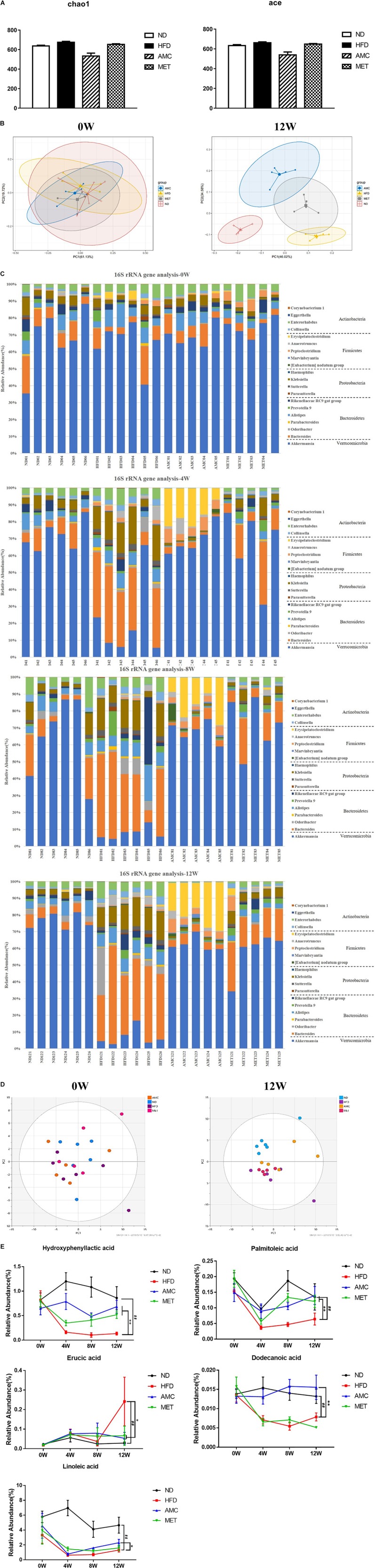
Dynamic alterations of fecal gut microbiota and gut microbial-host related co-metabolites across the lifespan in the study. **(A)** The estimators of α-diversity (chao 1, ace) of intestinal microbiota in each group at 12th week. **(B)** β-diversity reflected by PCA among ND group, HFD group, AMC group, and MET group at 0 week and 12th week. **(C)** Significantly changed genus of gut microbiota at week 0/4/8/12 in the different treated groups revealed by 16S rRNA gene sequencing (each color represents one bacterial genus). **(D)** The co-metabolites patterns of ND group, HFD group, AMC group, and MET group differentiated by PCA at 0 week and 12th week. **(E)** Significantly changed host-microbial co-metabolites after different intervention. Data are shown as means ± SEM of three independent experiments and were evaluated by ANOVA with Dunnett’s *post hoc* tests. ^#^*p* < 0.05, ^##^*p* < 0.01, compared with the ND group; *p < 0.05, **p < 0.01, compared with HFD group.

The α-diversity metrics (chao 1, ace) for different diets and treatments are presented in Figure 4A, with no significance among different groups. Then the β-diversity result of fecal bacterial communities of mice showed that, clustered based on principal component analysis (PCA), distinguished microbial communities based on diet and treatment protocol. As shown in Figure 4B, at 0 week, metagenomes of all mice groups clustered near each other. Excluding inter-individual differences, the initial composition of microbiota was similar among all groups. During the course of the experiment, as mice were introduced to different diet and treatment regimes, the fecal metagenomes of the HFD group diverged from that of the ND group. Moreover, fecal metagenomes of the AMC group formed a unique cluster compared to that derived from the untreated control HFD group. Thus, AMC treatment had a substantial effect on the gut microbial composition of HFD-fed mice.

Further analyses of fecal samples (weeks 0, 4, 8, and 12) at the genus level revealed significantly decreased proportion of sequences assigned to *Akkermansia* belonging to *Verrucomicrobia* in the metagenome of the HFD group compared to that of the ND group. However, the proportion of this bacterium was increased with AMC and MET treatment, as shown in Figure 4C. An opposite trend was observed in the expression of certain bacteria belonging to *Bacteroidetes* and *Proteobacteria*, including *Bacteroides*, *Odoribacter*, *Parabacteroides*, *Alistipes*, *Prevotella 9*, *Rikenellaceae RC9 gut group*, *Parasutterella*, *Klebsiella*, and *Haemophilus*. The proportion of certain beneficial microbial organisms, namely *Marvinbryantia*, *Peptoclostridium*, *Anaerotruncus*, and *Erysipelatoclostridium* were significantly enhanced in the metagenome of the AMC group compared to that of the HFD group. Finally, alterations in the primarily differentiated bacteria occurred from the 4th week of treatment and the distribution of the microbiota gradually stabilized with increased duration of the treatment period.

### AMC Treatment Induced Dynamic Changes in Gut Microbial-Host Related Co-metabolites

Gut microbial-host related co-metabolite analyses was performed on the fecal samples that were used for microbial analyses. The metabolites were identified by means of accurate mass measurements using GC-TOFMS and the data was compared with literature and/or database resources.

Initially, the main source of co-metabolite variation between the experimental groups at week 0 and 12 was analyzed using PCA (Figure 4D). Similar to PCA-based 16S rRNA gene analyses, excluding inter-individual differences, the initial co-metabolite composition was similar among all groups at week 0. However, during the course of the experiment, HFD and AMC treatment substantially affected the co-metabolites composition leading to divergence of groups.

Direct comparison of metabolites between different diet and treatment groups at week 0, 4, 8, and 12, identified five metabolites in the feces sample that may serve as potential biomarkers involved in the deregulation of gut microbiota (Figure 4E). Post-AMC treatment altered the gut metabolomics profile at week-4, -8, and -12 in the feces compared to that of the HFD group. Further, the significant decreases observed in the level of hydroxyphenyllactic acid (HPLA), palmitoleic acid, dodecanoic acid, and linoleic acid in the fecal samples of the HFD were partially recovered following AMC treatment. Furthermore, the level of erucic acid was significantly reduced in the feces of the AMC group compared to that of the HFD group, which was significantly higher than that of the ND group.

### Statistical Correlations

**FIGURE 5:**
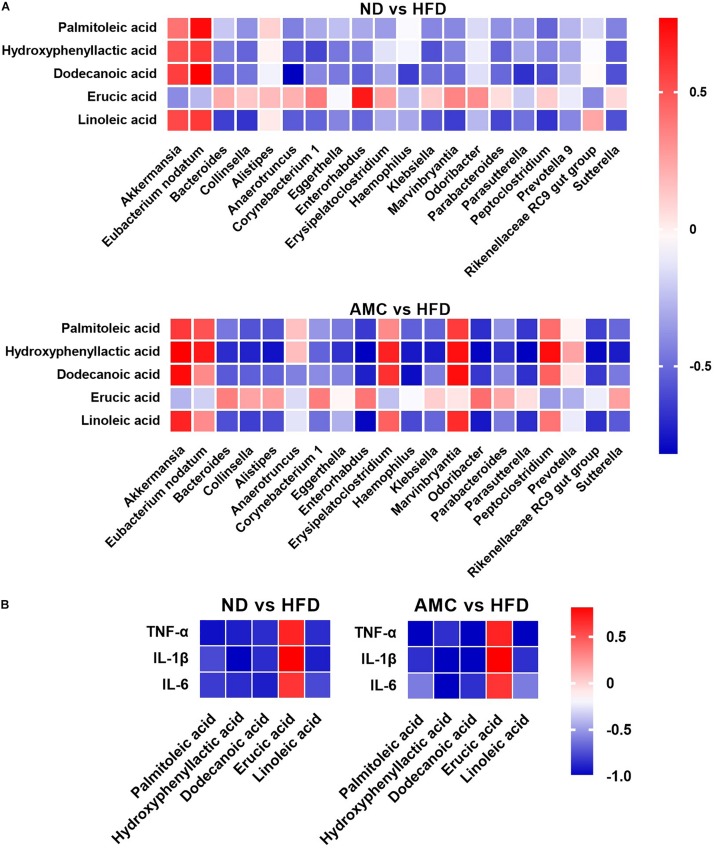
Heatmaps of Spearman correlation analysis. The correlation heatmap is used to represent significant statistical correlation values (*r*) between gut microbiota genera and altered fecal co-metabolites **(A)**, altered fecal co-metabolites and inflammatory cytokines **(B)** in ND group and HFD group, or HFD group and AMC group. In heatmap, red squares indicate significant positive correlations (*r* > 0.5, *p* < 0.05), white squares indicate non-significant correlations (*p* > 0.05), and blue squares indicate significant negative correlations (*r* < –0.5, *p* < 0.05). Data are shown as means ± SEM of three independent experiments and were evaluated by ANOVA with Dunnett’s *post hoc* tests. ^#^*p* < 0.05, ^##^*p* < 0.01, compared with the ND group; *p < 0.05, **p < 0.01, compared with HFD group.

Spearman correlation analysis was used to identify the potential link between altered gut microbiota composition and its association with fecal co-metabolites. Multiple significant associations were identified between the perturbed gut microbiota and altered fecal co-metabolites. Based on significant correlations (*r* > 0.5 or *r* < −0.5, *p* < 0.05) between the ND and HFD groups, and the HFD and AMC groups, *Akkermansia* was positively correlated with HPLA, dodecanoic acid, and linoleic acid levels. *Eubacterium nodatum* was positively correlated with palmitoleic acid and HPLA; while *Collinsella* was inversely correlated with HPLA and linoleic acid levels. *Bacteroides* displayed strong negative correlation with linoleic acid and HPLA was negatively correlated with *Klebsiella* and *Parabacteroides*. Lastly, dodecanoic acid was inversely associated with *Enterorhabdus*, *Haemophilus*, and *Parasutterella* (Figure 5A). For the potential connections between altered co-metabolites and inflammatory cytokines, inflammatory cytokines were inversely correlated with HPLA, palmitoleic acid, dodecanoic acid, and linoleic acid; while displayed positive correlation with erucic acid (Figure 5B).

## Discussion

In the present study, we combined the major active ingredients of the modified Gegen Qinlian decoction formula proven effective in treating T2DM and investigated the advantage of AMC on insulin resistance in HFD-induced T2DM mice *in vivo*. Metabolic disorders, such as adiposity, dyslipidemia, and T2DM induced by HFD are similar in animal models and humans (Ouwens et al., 2005; Kanakadurga et al., 2014). In our study, HFD-fed mice developed obesity and T2DM with impaired fasting glucose levels and increased body weight, body fat percentage, serum TC, and serum LDL-C levels. Similar to MET, a first-line drug used for the clinical treatment of T2DM, AMC treatment significantly reduced the general indicators of HFD-induced T2DM. These results showed that AMC served to improve the general indices of HFD-induced T2DM mice.

Insulin resistance is one of the most important determinants in T2DM pathogenesis and it may also induce the occurrence of other diseases (Patel et al., 2016; Saad et al., 2016; Neth and Craft, 2017). The effect of AMC on insulin resistance in mice with T2DM was investigated based on multiple perspectives. Our results from OGTT indicated significant AMC-induced glucose clearance capacity, suggesting the ability of AMC to improve glucose tolerance in HFD-fed mice. Further, the hyperinsulinemic-euglycemic clamp technique, the “gold standard” for evaluating insulin sensitivity (Lalia et al., 2015), confirmed the establishment of insulin resistance in C57BL/6J mice fed an HFD for 12 weeks, including excessive HGP, and establishment of a glucose uptake barrier in fat and muscle tissues, both of which were mitigated by AMC treatment, which significantly reduced insulin resistance in these peripheral target tissues. These findings were further confirmed by the GIR results, a quantitative indicator of insulin resistance. In addition, AMC treatment significantly enhanced the relative expression of InsR, IRS2, and phospho-Akt, which are required for activation of insulin signaling in the liver tissue. Taken together, these results demonstrate the ameliorative effect of AMC on glucose intolerance and insulin resistance in HFD-induced T2DM mice.

Obesity-related chronic inflammation is one of the initiating factors of insulin resistance (Xu L. et al., 2015; Almuraikhy et al., 2016); hence blocking inflammation would improve insulin sensitivity (Ghorpade et al., 2018). Specifically, the proinflammatory cytokine TNF-α has been reported to induce insulin resistance (Feinstein et al., 1993; Hotamisligil et al., 1993), and previous studies have established the role of inflammatory cytokines IL-1β and IL-6 in the development and progression of T2DM (Finucane et al., 2012; Mauer et al., 2015). The intestinal tract is an important immune organ, and the small intestine plays an important role in the pathogenesis of T2DM (Rubino, 2008). Our study showed that HFD, rather than ND, significantly induced the expression of TNF-α, IL-1β and IL-6 in the small intestinal tissue. However, treatment with both AMC and MET significantly reduced the expression of inflammatory cytokines, particularly TNF-α, to levels lower than those in the ND group, suggesting the ability of AMC to effectively alleviate intestinal inflammation.

Gut microbiota participates in the biological functions of the host in maintaining health, and forms a host-microbial cometabolism system, metabolizing dietary residues, drugs and other substances (Duffy et al., 2015; Ballet et al., 2018). Diet is the most important factor affecting the composition of intestinal flora (Chagwedera et al., 2019). Long-term administration of HFD leads to an imbalance in the structural order of intestinal flora causing chronic inflammation, leading to obesity, diabetes, coronary heart disease, and other chronic diseases (Sharma et al., 2018; Zou et al., 2018). In this study, fecal samples taken from the same source were subjected to both 16S sequencing and metabolite detection. Results of β-diversity of intestinal microbiota reflected by PCA revealed that specific diet and treatment regimens promoted major alterations in the gut microbiota and gut microbial-host related co-metabolites in mice. Furthermore, HFD significantly changed the structure of the community dynamics resulting in decreased abundance of *Akkermansia*, which is commonly recognized as a beneficial bacterium that is negatively associated with inflammation in the body (Bavineni et al., 2019). Further, *Akkermansia* supplementation alleviates obesity, inflammatory response in diabetic patients, insulin resistance, glucose tolerance, and other adverse symptoms. In the present study, both AMC and MET significantly enhanced the proportion of *Akkermansia*, suggesting the beneficial effects of AMC in treating T2DM. The ability of AMC to modulate the relative abundance of *Akkermansia* was consistent with previous results of clinical studies related to the Gegen Qinlian decoction (Xu J. et al., 2015). In addition, the abundance of certain bacteria was significantly reduced in the AMC group compared to that of the HFD group. These bacteria, including *Bacteroides* (Pedersen et al., 2016; Schulfer et al., 2018), *Odoribacter* (Schulfer et al., 2018), *Parabacteroides* (Kang et al., 2018), *Prevotella 9* (Pedersen et al., 2016), *Alistipes* (Kang et al., 2018; Yuan et al., 2018), *Parasutterella* (Chen et al., 2018), *Klebsiella* (Atarashi et al., 2017), and *Haemophilus* (Fernández-Calvet et al., 2018) were shown to positively correlate with the inflammatory state of the body, driving insulin resistance. Further, co-metabolite analyses identified significant AMC-induced metabolite changes in the feces that could serve as potential biomarkers in improving or inducing insulin resistance, including HPLA, palmitoleic acid, dodecanoic acid, linoleic acid, and erucic acid. These co-metabolites might also play a vital role in the regulation of metabolism.

Results from the correlation analyses indicated associations (*r* > 0.5 or *r* < −0.5, *p* < 0.05) between altered microbial communities and co-metabolites, co-metabolites and inflammatory cytokines. Specifically, *Akkermansia* is positively correlated with HPLA, dodecanoic acid, and linoleic acid, as *Akkermansia* was occupied certain proportions in ND and AMC group, these three significantly increased metabolites in AMC group are particularly noteworthy (*p* < 0.05, compared with HFD group). Meanwhile, HPLA, dodecanoic acid, and linoleic acid were inversely correlated with inflammatory cytokines, as well as evidences from other studies: HPLA inhibits the production of inflammatory factors (Aoki-Yoshida et al., 2013) and reduces inflammation in the body (Beloborodova et al., 2012), dodecanoic acid has been proven to have antibacterial and anti-inflammatory properties (Huang et al., 2014), linoleic acid normalizes inflammatory conditions associated with consumption of an HFD and reverses metabolic dysfunction in offspring (Segovia et al., 2015). Furthermore, HPLA was found to be negatively correlated with *Klebsiella* and *Parabacteroides* species. dodecanoic acid showed negative correlation with *Enterorhabdus*, *Haemophilus*, and *Parasutterella*, and *Bacteroides* exhibited strong negative correlation with linoleic acid. Taken together, these results indicate the participation of the anti-inflammatory effect of co-metabolites in the metabolic processes of the body in resisting inflammation, regulating impaired glucose tolerance, and improving insulin resistance to achieve the desired therapeutic effect in treating T2DM.

## Conclusion

In Conclusion, the present study supports the therapeutic use of AMC prepared from the major active ingredients of modified Gegen Qinlian decoction, a previously defined effective formula in treating T2DM. AMC significantly improved insulin resistance and alleviated the relevant indicators of T2DM by potentially modulating the bacteria-cometabolism-inflammatory responses.

## Data Availability Statement

The raw data supporting the conclusions of this article will be made available by the authors, without undue reservation, to any qualified researcher.

## Ethics Statement

All animal protocols were reviewed and approved by the Animal Ethics Committee of Nanjing Biomedical Research Institute of Nanjing University.

## Author Contributions

X-LT, and W-PJ conceived the study. H-TL and X-JZ assisted with the experimental design. LH and L-HZ planned and performed animal related experiments. M-LZ performed hyperinsulinemic-euglycemic clamp test. Z-ZG, X-MW, H-RW, and Y-JZ helped with experiments. X-TJ, Q-YD, and H-YY collected samples. LH analyzed all the data and wrote the manuscript. L-HZ reviewed the manuscript. All authors approved for final submission.

## Conflict of Interest

The authors declare that the research was conducted in the absence of any commercial or financial relationships that could be construed as a potential conflict of interest.
